# Importance of Photography Education to Improve Image Quality for Accurate Remote Diagnoses in Dental Trauma Patients: Observational Study

**DOI:** 10.2196/15152

**Published:** 2020-03-26

**Authors:** Jin-Sun Jeong, Nan-Sim Pang, Yiseul Choi, Kyeong-Mee Park, Taekbin Kim, Xin Xu, Wonse Park

**Affiliations:** 1 School and Hospital of Stomatology Shandong University Jinan China; 2 Shandong Key Laboratory of Oral Tissue Regeneration & Shandong Engineering Laboratory for Dental Materials and Oral Tissue Regeneration Jinan China; 3 Department of Advanced General Dentistry College of Dentistry Yonsei University Seoul Republic of Korea; 4 Human Identification Research Institute College of Dentistry Yonsei University Seoul Republic of Korea

**Keywords:** telemedicine, remote consultation, emergencies, tooth injuries, cell phone

## Abstract

**Background:**

High-quality photos are critical for the remote diagnosis of dental trauma and thus are beneficial to the prognosis. The quality of the images obtained using a cell phone depends on the level of dental and photography knowledge of the person who is taking the photos.

**Objective:**

This study aimed to determine the efficacy of photography education in improving images used for the remote diagnosis of dental trauma.

**Methods:**

The subjects comprised 30 laypeople and 30 dentists who were randomly assigned to 15 subgroups with 2 subjects in each. Each subject was asked to take photos of their own anterior teeth and those of their partner on the assumption that an accident occurred using both an iPhone 4s and iPhone 6. Education about how to take an appropriate photo of the anterior teeth for teleconsultation purposes was then provided, after which photos were taken again. Photos were assessed by a dentist for their usefulness in diagnosis.

**Results:**

This study analyzed 965 photos: 441 taken by laypeople and 524 taken by dentists. Photos taken after providing education had significantly higher scores for all assessment items than those taken before education (*P*<.05). The scores were also significantly higher for photos taken using the rear camera than those taken using the front camera (*P*<.02). The iPhone 6 did not have overwhelming advantages. The photos taken by dentists had significantly higher scores than those taken by laypeople for most of the evaluated items.

**Conclusions:**

Both laypeople and dentists might find photography education useful for when they are taking photos to be used in teleconsultations. The type of cell phone does not significantly affect the usefulness of such photos.

## Introduction

The World Health Organization has recently released a statement emphasizing the use of appropriate digital technologies for public health [1]. Ubiquitous health care (uHealth) services are rapidly developing due to increasing attention worldwide [2]. UHealth refers to combining information technology with medical services in order to provide remote medical and health management services that can be used anytime and anywhere [3]. UHealth allows for remote medical services such as remote examinations of and prescribing for various medical conditions as well as remote health management and enhancement services for healthy clients [4].

Rapid progress in information technology has resulted in successful implementation and testing of the electronic submission of clinical images for remote consultations in most medical and surgical subspecialties [5]. Moreover, implementations and research have been carried out using patient-to-doctor remote consultations, called telemedicine [6]. Home telenursing for patients suffering heart failure or diabetes, teleradiology for ultrasound and x-ray images, and teleconsultation for emergency orthopedic patients have been widely studied [7-12]. Studies related to dentistry have investigated remote medical procedures such as orthodontic consultations, remote oral care, preoperative evaluations before implant placement, and treatment of traumatic tooth injuries using teledentistry [13-16]. In cases of trauma to teeth or alveolar bone, the prognosis is most significantly affected by how rapidly a diagnosis is made and treatment is applied [17,18]. These considerations warrant the development of remote medical services allowing rapid diagnosis and treatment in the event of traumatic tooth injuries occurring when a dentist or other tooth injury expert is not available nearby.

Devices used to provide remote oral care services include oral video cameras (intraoral image capturing devices), digital single lens reflex (DSLR) cameras, and cell phones with built-in cameras [19-21]. An oral video camera is a small device that is easy to use but has the limitations of high-quality images being difficult to obtain due to its size and requiring a computer to display the images. In contrast, a DSLR camera allows for acquisition of images of exceptional quality, but it is relatively expensive, requires special imaging equipment, and the operator needs to be trained; hence, it is not an optimal imaging device for emergency or remote consultations.

Cell phones are highly portable, have high-resolution cameras, and are now almost ubiquitously used by people of all age groups. Continuing developments have allowed for such devices to be used not only for telecommunication but also to provide multiple computer-like communication functions including text, photo, and video transfer, as well as internet access. Different types of remote consultation or treatment based on the use of cell phones are currently being investigated in the field of telemedicine [22,23]. For teleconsultations, cell phone cameras are better than DSLR and oral video cameras in terms of convenience and portability [24].

However, the quality of the images obtained using a cell phone depends on the level of dental knowledge of the person who is taking the photos and the imaging conditions [16]. Zaror et al [25] studied the efficacy of an app for traumatic dental injuries and the quality of images for clinical purposes, but photography education was not involved. An in vitro study identified several important camera-related factors that could influence the image quality for teledentistry in dentoalveolar trauma, including autofocusing and antimovement functions, and the automatic white-balance function was helpful for detecting the color area [26].

This study investigated differences in image quality according to who photographed the traumatized tooth (dentist vs laypeople), the effect of receiving photography education (before vs after), and what model cell phone was used (including different numbers of camera pixels).

## Methods

### Recruitment

This clinical research was approved by the institutional review board of the dental hospital at Yonsei University (number 2-2012-0025). The subjects consisted of 60 Korean adults (30 laypeople and 30 dentists) selected from 62 volunteers. The laypeople comprised 21 males and 9 females ranging in age from 21 to 39 years (28.67 [SD 4.20] years), while the dentists comprised 14 males and 16 females who ranged in age from 26 to 37 years (30.74 [SD 3.21] years). The inclusion criteria were being older than 20 years, able to operate the camera on a cell phone, having continuous anterior dentition (including any prosthesis and orthodontic brackets), and signing the consent form for the experiment.

**Figure 1:**
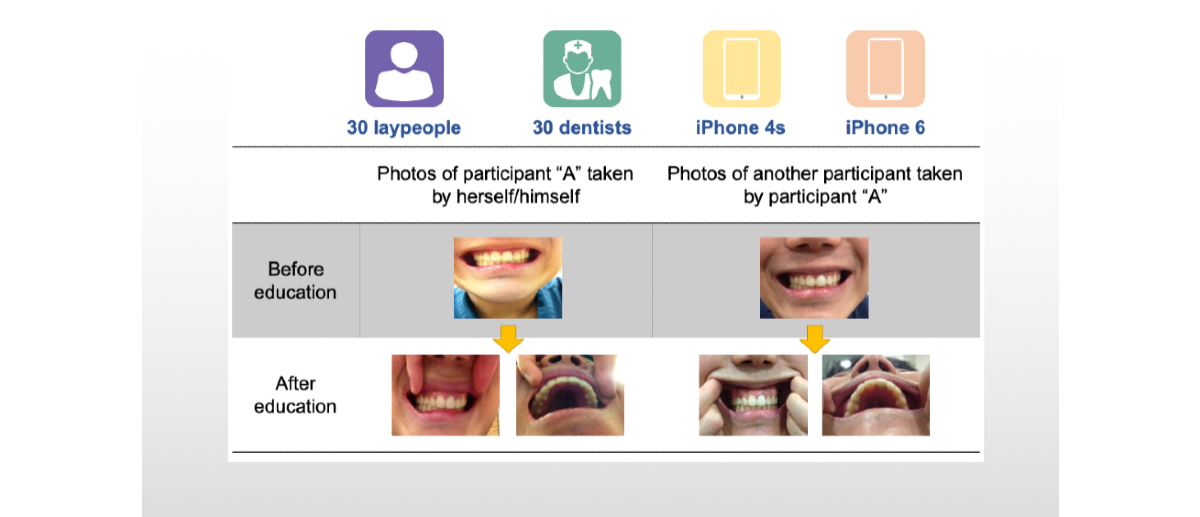
Summary of experimental design.

The subjects were randomly arranged into 15 subgroups with 2 subjects each, and they were asked to take photos of themselves and their partner using both an iPhone 4s (0.3-megapixel front camera, 8-megapixel rear camera, Apple), and iPhone 6 (1.2-megapixel front camera, 8-megapixel rear camera, Apple) before and after receiving photography education (Figure 1). There was no limit to the number of photos each subject could take.

### Experimental Design

We assumed that injuries had occurred in the subjects because of an accident. Subjects were encouraged to freely take photos of the maxillary anterior area including 4 incisors and 2 canines by using the functions of autofocusing, blurring, white balance, focal length, and resolution provided by the cell phone, with the built-in front camera and the default camera app. They were then asked to take photos of their colleagues’ teeth using the same methods but with the built-in rear camera.

Photography education was then provided by an experienced dental hygienist, face to face, to every subject with the following contents: how to take photos from frontal and occlusal views, how to take a photo in which all 6 anterior teeth appear, how to use a retractor to expose soft tissue, how to adjust the focus, and how to adjust the camera settings according to the protocol proposed by Park et al [26] to better exhibit the anatomy and discoloration of the teeth as well as the gingival texture. The education session lasted about 15 minutes, and the subjects were then asked to repeat the experiment following the instructions provided in the photography education.

### Photo Assessments

The captured images were assessed twice with a 1-week interval on a desktop computer (LG, screen resolution 1920×1080 pixels, 8-bit color depth) by a single dentist in the Department of Advanced General Dentistry at Yonsei University.

**Figure 2:**
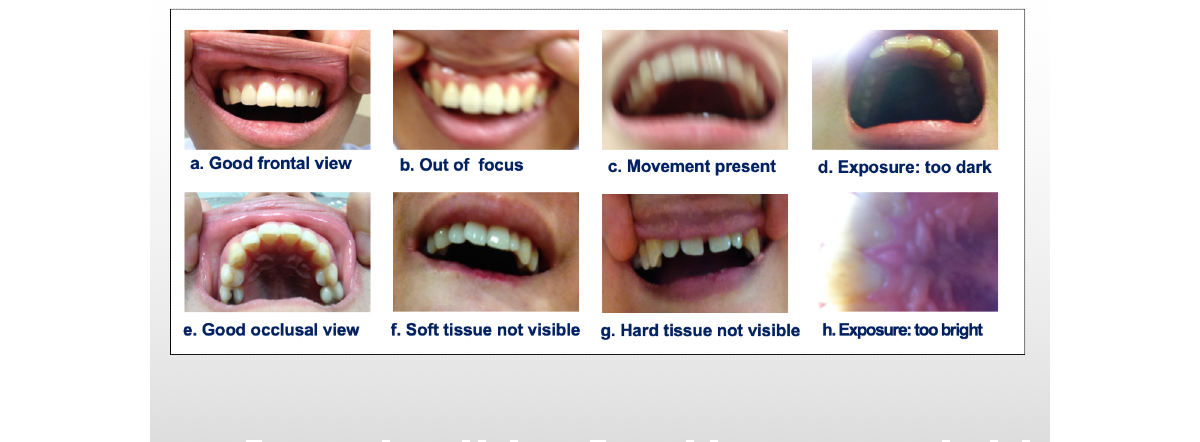
Examples of photos that were appropriate and inappropriate for the initial assessment.

After all the photos had been collected, errors that appeared frequently during the experiment were analyzed and images of frontal and occlusal views were evaluated. Frequent errors included retraction failure and incomplete frontal and occlusal views. Each photo was evaluated as either good or failure, and the relationships between this categorization and the frequency of errors were evaluated by the dentist (Figure 2). The photos evaluated as good were scored on the following 5-point numerical scale: 1=not suitable for making a diagnosis, 2=able to make a questionable diagnosis, 3=able to make an average diagnosis, 4=able to make a good diagnosis, and 5=able to make a perfect diagnosis.

The two assessments meant that the assessment score for each tooth type was on a 10-point scale. The highest score was used when multiple photos of the same type were assessed. The central incisors, lateral incisors, and canines were assessed separately against the items listed in Table 1 for soft tissue and hard tissue to produce a final assessment score of 30 points.

**Table 1:** Assessment items.

Category and finding			Suggested situation
**General**				
		Optimal focus	—
		Movement present	—
		Exposure	—
**Hard tissue**				
	**Frontal**			
		Shape	Crown fracture
		Position	Displacement
		Alignment	Displacement
		Bleeding spot with pink color	Pulp exposure
	**Occlusal**			
		Shape	Crown fracture
		Position	Displacement
		Alignment	Displacement
		Bleeding spot with pink color	Pulp exposure
**Soft tissue**				
	**Frontal**			
		Gingival sulcus	Gingival bleeding
		Integrity	Laceration
		Color	Hematoma
	**Occlusal**			
		Gingival sulcus	Gingival bleeding
		Integrity	Laceration
		Color	Hematoma

### Statistical Analysis

Data were analyzed using SPSS Statistics version 25.0 (IBM Corporation). The intrarater reliability was assessed using Cohen kappa. Paired *t* tests were performed to compare photo quality between the two models of cell phone (iPhone 4s and iPhone 6) and between before and after the photography education. Two-sample Student *t* tests were used to compare the photo quality between laypeople and dentists and between the photos taken of oneself and the subgroup partner.

## Results

Each photo was assessed by the dentist twice. The kappa indexes for intrarater reliability were .764 and .728 for the general assessment and the assessments of hard tissue and soft tissue, respectively.

This study analyzed 965 photos: 441 taken by laypeople and 524 taken by dentists. Frontal view photos were taken by all subjects before and after receiving education. In contrast, occlusal area photos were taken by only 16 subjects (4 laypeople and 12 dentists) which were further taken by all subjects after education, and so these photos were not suitable for performing comparisons.

In the group of laypeople, photos in which the retraction was evaluated as being appropriate were taken by 8 subjects with the front camera and 12 with the rear camera by the partners before receiving education, increasing to 23 and 28, respectively, after receiving education. In the group of dentists, photos in which the retraction was evaluated as being appropriate were taken by 20 subjects with the front camera and 19 with the rear camera by the partners before receiving education, increasing to 28 and 30, respectively, after receiving education.

Photos taken after education had significantly higher scores than those taken before education with the exception of the evaluation item of optimal focus for the iPhone 6 front camera (Multimedia Appendix 1). Photos taken using the rear camera had significantly higher scores than those taken using the front camera by oneself (Multimedia Appendix 2). Although photos taken using the rear camera of the iPhone 6 had significantly higher scores in some items than those taken using the iPhone 4s, the iPhone 6 did not have overwhelming advantages (Multimedia Appendix 3). Photos taken by dentists had significantly higher scores than those taken by laypeople for most of the evaluated items (Multimedia Appendix 4).

## Discussion

### Principal Results

This study assessed the use of cell phones for teleconsultations in dentistry. It was found that the photo scores were significantly higher for those taken by dentists than by laypeople for most of the evaluated items and for those taken after receiving education compared with beforehand except for one item in the iPhone 6 group with the front camera. The iPhone 6 did not have overwhelming advantages over the iPhone 4s.

There were common errors observed in the photos. Frontal view photos—and not occlusal view photos—were taken by both laypeople and dentists, and many images were taken without appropriate retraction. However, after participants received instruction in photography, the error rates in both the layperson and dentist groups were markedly reduced. Moreover, there were statistically significant differences in most of the evaluation categories between before and after receiving the education. These observations support the usefulness of providing image-taking instructions to both laypeople and medical staff in photography education sessions on appropriate methods for dental trauma teleconsultation using cell phones.

While the dentist group received higher evaluation scores both before and after the education compared with the laypeople group, there was no significant intergroup difference in the score for the item of shape in occlusal photos of hard tissue after the education using either the iPhone 4s or iPhone 6 (*P*>.05). These findings suggest that the laypeople can benefit from receiving education about how to take photos for use in dental evaluations, although not to the extent of dentists who have professional knowledge of dentistry. Comparisons based on the numbers of pixels of the camera showed that images taken with the rear camera scored higher than images taken with the front camera. For self-images taken using the front camera, images taken with the iPhone 4s (0.3 megapixels) had higher evaluation scores than images taken with the iPhone 6 (1.2 megapixels). This surprising result is probably due to factors other than the number of pixels, such as the size and weight of the device and the grip sensitivity. Moreover, the rear camera of a cell phone generally has a higher resolution than the front camera, and the rear cameras of both the iPhone 4s and iPhone 6 have 8.0 megapixels. However, the images taken with the iPhone 6 received higher scores probably because of its superior image sensor.

### Comparison With Previous Work

According to the in vitro study of Park et al [26], autofocusing and white balance play important roles in photos taken using a cell phone for teleconsultation purposes. Similarly, our study found that focus, movement, and exposure characteristics affected the image quality. Another study using the same protocol as Park found that the precision of remote diagnoses was comparable to diagnoses conducted in person for photos that were taken by dentists [27].

There are several reports of satisfactory results being obtained when using cell phone apps for caries screening and traumatic dental injuries [25,28,29]. An intentionally developed cell phone app for traumatic dental injuries was recently evaluated for validity and usability [25]. That app allowed users to select from a library of images of the most common injuries and so could be used by any person regardless of their level of knowledge about dental trauma. However, its accuracy as well as the quality of the information reported under emergency conditions should be evaluated in the future.

In a study of the usefulness of an app in diagnosing dental trauma, Mohan et al [30] concluded that photos of injured teeth and soft tissue can be used by dentists to give a correct diagnosis. This indicates that a cell phone app that allows teleconsultation using the in-built cameras should be developed and used. The end goals of such a new app would be (1) to allow medical staff with minimal experience of dental trauma (ie, school nurses and emergency room medical staff) to send images of patients to dental trauma experts in real time and also receive a remote diagnosis and information about appropriate first aid treatment in real time and (2) for laypeople to be able to directly use the app and receive consultations to facilitate remote diagnosis and treatment via image transfer if a dentist is not available. In summary, the app should initially provide the basic functions of teleconsultation, and other additional functions should be theoretically grounded and evidence-based [31]. The International Association for Dental Traumatology provides a publicly available first aid protocol for dental trauma patients that patients can download from the internet to educate or treat themselves [32-34]. If an app that supports teleconsultation is developed, an additional protocol for performing an accurate diagnosis (ie, the imaging protocol suggested in our study) could be added to the instructions for patients.

However, a cell phone camera should be viewed as a complementary tool for use in teleconsultation, since images of dental trauma patients taken using cell phones may be different from images taken at a dental clinic. Moumoulidis et al [35] suggested that telemedicine does not always facilitate correct physician assessments, since their clinical trial found that 62% of diagnoses of nasal fractures based on images from cell phone cameras did not agree with the clinical assessments. This highlights that remote teleconsultations performed using cell phones do not guarantee accurate diagnoses, and so apps should be limited to use as a complementary tool to allow for appropriate emergency first aid in dental emergencies. Moreover, patients who receive teleconsultations should also seek appropriate treatment by visiting a dental clinic as soon as possible.

### Limitations

This study was subject to several limitations. First, the study involved healthy subjects rather than actual trauma patients. Second, instead of evaluating images that had been taken with a cell phone and then transferred, the imaged files were analyzed directly on a computer screen. Third, we used iPhone 4s and iPhone 6 cell phones for the teleconsultations since most related studies have used iPhone devices, and so it might be worthwhile to repeat the experiment using Android cell phones in the future. Future studies should address the limitations of this study including trying to mimic actual traumatic events.

### Conclusions

Photography education is effective for both laypeople and dentists. Further developments of teleconsultation using the camera built into a cell phone require an optimal photo-taking protocol that should include the following factors: (1) obtaining frontal and occlusal images with proper retraction applied so that 6 anterior teeth and soft tissue are clearly visible, (2) photos should be taken by another person, and (3) the rear rather than the front camera should be used in order to optimize the image quality.

## Appendix

Multimedia Appendix 1 Comparison of the quality of photos taken before and after education.

Multimedia Appendix 2 Comparison of the quality of photos taken using front and rear cameras.

Multimedia Appendix 3 Comparison of the quality of photos taken with the iPhone 4s and iPhone 6.

Multimedia Appendix 4 Comparison of the quality of photos taken by laypeople and dentists.

## Abbreviations

uHealth: ubiquitous health care
DSLR: digital single lens reflex
