# Supplementary material for: Importance of Photography Education to Improve Image Quality for Accurate Remote Diagnoses in Dental Trauma Patients: Observational Study
Source: JMIR Mhealth Uhealth. 2020 Mar 26;8(3):e15152. doi: 10.2196/15152 (PMC7146236; doi:10.2196/15152)
Supplement: Multimedia Appendix 1 [file mhealth_v8i3e15152_app1.docx]

Multimedia Appendix 1. Comparison of the quality of photos taken before and after education.

|  | |  |  | Front camera of iPhone 4s | | |  | Rear camera of iPhone 4s | | |  | Front camera of iPhone 6 | | |  | Rear camera of iPhone 6 | | |  |
| --- | --- | --- | --- | --- | --- | --- | --- | --- | --- | --- | --- | --- | --- | --- | --- | --- | --- | --- | --- |
| Category | | | Finding | Before education | After education | *P* |  | Before education | After education | *P* |  | Before education | After education | *P* |  | Before education | After education | *P* |  |
| General | | | Optimal focus | 5.45 | 5.77 | .023^a^ |  | 6.87 | 7.93 | <.001^a^ |  | 5.07 | 5.37 | .063 |  | 7.62 | 8.52 | <.001^a^ |  |
|  |  |  | Movement present | 5.63 | 6.42 | <.001^a^ |  | 7.02 | 8.02 | <.001^a^ |  | 5.43 | 6.00 | .002^a^ |  | 7.60 | 8.67 | <.001^a^ |  |
|  |  |  | Exposure | 5.57 | 6.03 | .001^a^ |  | 6.85 | 7.78 | <.001^a^ |  | 5.45 | 5.82 | .016^a^ |  | 7.03 | 7.87 | <.001^a^ |  |
| Hard  tissue | Frontal | | Shape | 14.63 | 16.87 | <.001^a^ |  | 19.47 | 22.78 | <.001^a^ |  | 14.32 | 15.70 | .005^a^ |  | 21.60 | 24.78 | <.001^a^ |  |
|  |  |  | Position | 17.23 | 19.23 | <.001^a^ |  | 20.92 | 25.53 | <.001^a^ |  | 16.75 | 18.32 | .002^a^ |  | 23.10 | 26.50 | <.001^a^ |  |
|  |  |  | Alignment | 18.92 | 21.25 | <.001^a^ |  | 23.10 | 26.73 | <.001^a^ |  | 18.85 | 20.25 | .009^a^ |  | 24.85 | 27.80 | <.001^a^ |  |
|  |  |  | Bleeding spot with pink color | 11.37 | 14.20 | <.001^a^ |  | 16.18 | 19.75 | <.001^a^ |  | 10.97 | 12.93 | <.001^a^ |  | 18.52 | 22.35 | <.001^a^ |  |
|  | Occlusal | | Shape | N/A | N/A |  |  | N/A | N/A |  |  | N/A | N/A |  |  | N/A | N/A |  |  |
|  |  |  | Position | N/A | N/A |  |  | N/A | N/A |  |  | N/A | N/A |  |  | N/A | N/A |  |  |
|  |  |  | Alignment | N/A | N/A |  |  | N/A | N/A |  |  | N/A | N/A |  |  | N/A | N/A |  |  |
|  |  |  | Bleeding spot with pink color | N/A | N/A |  |  | N/A | N/A |  |  | N/A | N/A |  |  | N/A | N/A |  |  |
| Soft  tissue | Frontal | | Gingival sulcus | 11.13 | 15.80 | <.001^a^ |  | 14.53 | 23.20 | <.001^a^ |  | 10.85 | 14.40 | <.001^a^ |  | 16.22 | 24.47 | <.001^a^ |  |
|  |  |  | Integrity | 9.75 | 15.25 | <.001^a^ |  | 12.78 | 22.83 | <.001^a^ |  | 9.77 | 14.17 | <.001^a^ |  | 14.18 | 24.70 | <.001^a^ |  |
|  |  |  | Color | 9.75 | 15.30 | <.001^a^ |  | 12.65 | 22.73 | <.001^a^ |  | 9.77 | 14.17 | <.001^a^ |  | 13.98 | 24.35 | <.001^a^ |  |
|  | Occlusal | | Gingival sulcus | N/A | N/A |  |  | N/A | N/A |  |  | N/A | N/A |  |  | N/A | N/A |  |  |
|  |  |  | Integrity | N/A | N/A |  |  | N/A | N/A |  |  | N/A | N/A |  |  | N/A | N/A |  |  |
|  |  |  | Color | N/A | N/A |  |  | N/A | N/A |  |  | N/A | N/A |  |  | N/A | N/A |  |  |
| ^a^ Statistically significant in bivariate comparison between before and after education (*P* < .05 in paired *t*-test). | | | | | | | | | | | | | | | | | | |  |
| N/A, data not applicable to comparison. | | | | | | | | | | | | | | | | | | |  |
