# Supplementary material for: Importance of Photography Education to Improve Image Quality for Accurate Remote Diagnoses in Dental Trauma Patients: Observational Study
Source: JMIR Mhealth Uhealth. 2020 Mar 26;8(3):e15152. doi: 10.2196/15152 (PMC7146236; doi:10.2196/15152)
Supplement: Multimedia Appendix 2 [file mhealth_v8i3e15152_app2.docx]

Multimedia Appendix 2. Comparison of the quality of photos taken using front and rear cameras.

|  | |  |  | Photos taken with iPhone 4s before education | | |  | Photos taken with iPhone 4s  after education | | |  | Photos taken with iPhone 6 before education | | |  | Photos taken with iPhone 6 after education | | |  |
| --- | --- | --- | --- | --- | --- | --- | --- | --- | --- | --- | --- | --- | --- | --- | --- | --- | --- | --- | --- |
| Category | | | Finding | Front camera | Rear camera | *P* |  | Front  camera | Rear camera | *P* |  | Front camera | Rear camera | *P* |  | Front  camera | Rear camera | *P* |  |
| General | | | Optimal focus | 5.45 | 6.87 | <.001^a^ |  | 5.77 | 7.93 | <.001^a^ |  | 5.07 | 7.62 | <.001^a^ |  | 5.33 | 8.52 | <.001^a^ |  |
|  |  |  | Movement present | 5.63 | 7.02 | <.001^a^ |  | 6.42 | 8.30 | <.001^a^ |  | 5.43 | 7.67 | <.001^a^ |  | 5.90 | 8.67 | <.001^a^ |  |
|  |  |  | Exposure | 5.57 | 6.85 | <.001^a^ |  | 6.03 | 7.82 | <.001^a^ |  | 5.38 | 7.08 | <.001^a^ |  | 5.77 | 7.87 | <.001^a^ |  |
| Hard tissue | Frontal | | Shape | 14.63 | 19.47 | <.001^a^ |  | 16.87 | 22.78 | <.001^a^ |  | 14.32 | 21.60 | <.001^a^ |  | 15.70 | 24.78 | <.001^a^ |  |
|  |  |  | Position | 17.23 | 20.92 | <.001^a^ |  | 19.23 | 25.53 | <.001^a^ |  | 16.75 | 23.10 | <.001^a^ |  | 18.32 | 26.50 | <.001^a^ |  |
|  |  |  | Alignment | 18.92 | 23.10 | <.001^a^ |  | 21.25 | 26.73 | <.001^a^ |  | 18.85 | 24.85 | <.001^a^ |  | 20.25 | 27.80 | <.001^a^ |  |
|  |  |  | Bleeding spot with pink color | 11.37 | 16.18 | <.001^a^ |  | 14.20 | 19.75 | <.001^a^ |  | 10.97 | 18.52 | <.001^a^ |  | 12.93 | 22.35 | <.001^a^ |  |
|  | Occlusal | | Shape | N/A | N/A |  |  | 12.60 | 18.55 | <.001^a^ |  | N/A | N/A |  |  | 12.37 | 19.00 | <.001^a^ |  |
|  |  |  | Position | N/A | N/A |  |  | 14.95 | 21.10 | <.001^a^ |  | N/A | N/A |  |  | 14.33 | 21.13 | <.001^a^ |  |
|  |  |  | Alignment | N/A | N/A |  |  | 16.60 | 22.43 | <.001^a^ |  | N/A | N/A |  |  | 16.58 | 23.60 | <.001^a^ |  |
|  |  |  | Bleeding spot with pink color | N/A | N/A |  |  | 9.28 | 15.43 | <.001^a^ |  | N/A | N/A |  |  | 9.95 | 15.53 | <.001^a^ |  |
| Soft tissue | Frontal | | Gingival sulcus | 11.13 | 14.53 | .002^a^ |  | 15.80 | 23.20 | <.001^a^ |  | 10.85 | 16.22 | <.001^a^ |  | 14.40 | 24.47 | <.001^a^ |  |
|  |  |  | Integrity | 9.75 | 12.78 | .014^a^ |  | 15.25 | 22.83 | <.001^a^ |  | 9.77 | 14.18 | .002^a^ |  | 14.17 | 24.70 | <.001^a^ |  |
|  |  |  | Color | 9.75 | 12.65 | .018^a^ |  | 15.30 | 22.73 | <.001^a^ |  | 9.77 | 13.98 | .003^a^ |  | 14.17 | 24.35 | <.001^a^ |  |
|  | Occlusal | | Gingival sulcus | N/A | N/A |  |  | 8.52 | 15.43 | <.001^a^ |  | N/A | N/A |  |  | 9.42 | 15.33 | <.001^a^ |  |
|  |  |  | Integrity | N/A | N/A |  |  | 8.83 | 16.00 | <.001^a^ |  | N/A | N/A |  |  | 9.53 | 16.30 | <.001^a^ |  |
|  |  |  | Color | N/A | N/A |  |  | 8.98 | 15.90 | <.001^a^ |  | N/A | N/A |  |  | 9.58 | 16.35 | <.001^a^ |  |
| ^a^ Statistically significant in bivariate comparison between the front camera and rear camera (P < .05 in two-sample Student’s *t*-test). | | | | | | | | | | | | | | | | | | |  |
