# Supplementary material for: Importance of Photography Education to Improve Image Quality for Accurate Remote Diagnoses in Dental Trauma Patients: Observational Study
Source: JMIR Mhealth Uhealth. 2020 Mar 26;8(3):e15152. doi: 10.2196/15152 (PMC7146236; doi:10.2196/15152)
Supplement: Multimedia Appendix 3 [file mhealth_v8i3e15152_app3.docx]

Multimedia Appendix 3. Comparison of the quality of photos taken with the iPhone 4s and iPhone 6.

|  |  |  | Photos taken using front camera before education | | |  | Photos taken using rear camera before education | | |  | Photos taken using front camera after education | | |  | Photos taken using rear camera after education | | |  |
| --- | --- | --- | --- | --- | --- | --- | --- | --- | --- | --- | --- | --- | --- | --- | --- | --- | --- | --- |
| Category | | Finding | iPhone 4s | iPhone 6 | *P* |  | iPhone 4s | iPhone 6 | *P* |  | iPhone 4s | iPhone 6 | *P* |  | iPhone 4s | iPhone 6 | *P* |  |
| General | | Optimal focus | 5.45 | 5.07 | .008^a^ |  | 6.87 | 7.62 | .006^a^ |  | 5.77 | 5.37 | .015^a^ |  | 7.93 | 8.52 | .015^a^ |  |
|  |  | Movement present | 5.63 | 5.43 | .141 |  | 7.02 | 7.60 | .017^a^ |  | 6.42 | 6.00 | .025^a^ |  | 8.20 | 8.67 | .050 |  |
|  |  | Exposure | 5.57 | 5.45 | .441 |  | 6.85 | 7.03 | .300 |  | 6.03 | 5.82 | .274 |  | 7.78 | 7.87 | .682 |  |
| Hard  tissue | Frontal | Shape | 14.63 | 14.32 | .464 |  | 19.47 | 21.60 | .003^a^ |  | 16.87 | 15.70 | .025^a^ |  | 22.78 | 24.78 | .007^a^ |  |
|  |  | Position | 17.23 | 16.75 | .260 |  | 20.92 | 23.10 | .001^a^ |  | 19.23 | 18.32 | .079 |  | 25.53 | 26.50 | .100 |  |
|  |  | Alignment | 18.92 | 18.85 | .885 |  | 23.10 | 24.85 | .005^a^ |  | 21.25 | 20.25 | .094 |  | 26.73 | 27.80 | .086 |  |
|  |  | Bleeding spot with pink color | 11.37 | 10.97 | .363 |  | 16.18 | 18.52 | .002^a^ |  | 14.20 | 12.93 | .018^a^ |  | 19.75 | 22.35 | .002^a^ |  |
|  | Occlusal | Shape | N/A | N/A |  |  | N/A | N/A |  |  | 12.60 | 12.37 | .659 |  | 18.55 | 19.00 | .516 |  |
|  |  | Position | N/A | N/A |  |  | N/A | N/A |  |  | 14.95 | 14.33 | .273 |  | 21.10 | 21.13 | .957 |  |
|  |  | Alignment | N/A | N/A |  |  | N/A | N/A |  |  | 16.60 | 16.58 | .982 |  | 22.43 | 23.60 | .060 |  |
|  |  | Bleeding spot with pink color | N/A | N/A |  |  | N/A | N/A |  |  | 9.28 | 9.95 | .180 |  | 15.43 | 15.53 | .867 |  |
| Soft  tissue | Frontal | Gingival sulcus | 11.13 | 10.85 | .539 |  | 14.53 | 16.22 | .027^a^ |  | 15.80 | 14.40 | .027^a^ |  | 23.20 | 24.47 | .102 |  |
|  |  | Integrity | 9.75 | 9.77 | .973 |  | 12.78 | 14.18 | .044^a^ |  | 15.25 | 14.17 | .118 |  | 22.83 | 24.70 | .017^a^ |  |
|  |  | Color | 9.75 | 9.77 | .973 |  | 12.65 | 13.98 | .054 |  | 15.30 | 14.17 | .095 |  | 22.73 | 24.35 | .037^a^ |  |
|  | Occlusal | Gingival sulcus | N/A | N/A |  |  | N/A | N/A |  |  | 8.52 | 9.42 | .094 |  | 15.43 | 15.33 | .884 |  |
|  |  | Integrity | N/A | N/A |  |  | N/A | N/A |  |  | 8.83 | 9.53 | .209 |  | 16.00 | 16.30 | .650 |  |
|  |  | Color | N/A | N/A |  |  | N/A | N/A |  |  | 8.98 | 9.58 | .278 |  | 15.90 | 16.35 | .476 |  |
| ^a^ Statistically significant in bivariate comparison between iPhone 4s and iPhone 6 (*P* < .05 in paired *t*-test). | | | | | | | | | | | | | | | | | | |
