# Supplementary material for: Importance of Photography Education to Improve Image Quality for Accurate Remote Diagnoses in Dental Trauma Patients: Observational Study
Source: JMIR Mhealth Uhealth. 2020 Mar 26;8(3):e15152. doi: 10.2196/15152 (PMC7146236; doi:10.2196/15152)
Supplement: Multimedia Appendix 4 [file mhealth_v8i3e15152_app4.docx]

Multimedia Appendix 4. Comparison of the quality of photos taken by laypeople and dentists.

|  | |  |  | Photos taken with iPhone 4s before education | | |  | Photos taken with iPhone 4s after education | | |  | Photos taken with iPhone 6 before education | | |  | Photos taken with iPhone 6 after education | | |  |
| --- | --- | --- | --- | --- | --- | --- | --- | --- | --- | --- | --- | --- | --- | --- | --- | --- | --- | --- | --- |
| Category | | | Finding | Laypeople | Dentists | *P* |  | Laypeople | Dentists | *P* |  | Laypeople | Dentists | *P* |  | Laypeople | Dentists | *P* |  |
| General | | | Optimal focus | 5.52 | 6.80 | <.001^a^ |  | 6.35 | 7.35 | .002^a^ |  | 5.83 | 6.85 | .004^a^ |  | 6.47 | 7.38 | .021^a^ |  |
|  |  |  | Movement present | 5.58 | 7.07 | <.001^a^ |  | 6.73 | 7.98 | <.001^a^ |  | 5.97 | 7.13 | <.001^a^ |  | 6.72 | 7.85 | .002^a^ |  |
|  |  |  | Exposure | 5.60 | 6.82 | <.001^a^ |  | 6.38 | 7.47 | <.001^a^ |  | 5.55 | 6.92 | <.001^a^ |  | 6.33 | 7.30 | .001^a^ |  |
| Hard tissue | Frontal | | Shape | 14.90 | 19.20 | <.001^a^ |  | 18.15 | 21.50 | .001^a^ |  | 16.30 | 19.62 | .001^a^ |  | 19.25 | 21.23 | .079 |  |
|  |  |  | Position | 16.80 | 21.35 | <.001^a^ |  | 15.75 | 19.20 | .039^a^ |  | 17.45 | 22.40 | <.001^a^ |  | 20.38 | 24.43 | <.001^a^ |  |
|  |  |  | Alignment | 18.83 | 23.18 | <.001^a^ |  | 21.88 | 26.10 | <.001^a^ |  | 19.45 | 24.25 | <.001^a^ |  | 22.57 | 25.48 | .006^a^ |  |
|  |  |  | Bleeding spot with pink color for | 11.65 | 15.90 | <.001^a^ |  | 15.25 | 18.70 | .002^a^ |  | 12.78 | 16.70 | .001^a^ |  | 16.55 | 18.73 | .075 |  |
|  | Occlusal | | Shape | N/A | N/A |  |  | 14.83 | 16.32 | .107 |  | N/A | N/A |  |  | 14.88 | 16.48 | .088 |  |
|  |  |  | Position | N/A | N/A |  |  | 16.00 | 20.05 | <.001^a^ |  | N/A | N/A |  |  | 16.13 | 19.33 | .001^a^ |  |
|  |  |  | Alignment | N/A | N/A |  |  | 17.68 | 21.35 | .001^a^ |  | N/A | N/A |  |  | 18.37 | 21.82 | .002^a^ |  |
|  |  |  | Bleeding spot with pink color | N/A | N/A |  |  | 10.95 | 13.77 | .002^a^ |  | N/A | N/A |  |  | 11.48 | 14.00 | .003^a^ |  |
| Soft tissue | Frontal | | Gingival sulcus | 10.53 | 15.13 | <.001^a^ |  | 16.60 | 22.40 | <.001^a^ |  | 11.57 | 15.50 | .003^a^ |  | 17.32 | 21.55 | .002^a^ |  |
|  |  |  | Integrity | 8.62 | 13.92 | <.001^a^ |  | 15.78 | 22.30 | <.001^a^ |  | 9.55 | 14.40 | .001^a^ |  | 16.52 | 22.35 | <.001^a^ |  |
|  |  |  | Color | 8.42 | 13.98 | <.001^a^ |  | 15.63 | 22.40 | <.001^a^ |  | 9.50 | 14.25 | .001^a^ |  | 16.27 | 22.25 | <.001^a^ |  |
|  | Occlusal | | Gingival sulcus | N/A | N/A |  |  | 10.68 | 13.27 | .009^a^ |  | N/A | N/A |  |  | 10.90 | 13.85 | .001^a^ |  |
|  |  |  | Integrity | N/A | N/A |  |  | 10.92 | 13.92 | .004^a^ |  | N/A | N/A |  |  | 11.57 | 14.27 | .008^a^ |  |
|  |  |  | Color | N/A | N/A |  |  | 10.97 | 13.92 | .003^a^ |  | N/A | N/A |  |  | 11.62 | 14.32 | .008^a^ |  |
| ^a^ Statistically significant in bivariate comparison between laypeople and dentists (*P* < .05 in two-sample Student’s *t*-test). | | | | | | | | | | | | | | | | | | |  |
